# Development of a Field-Deployable Loop-Mediated Isothermal Amplification Assay for the Rapid Detection of *Erysiphe corylacearum* in Hazelnut

**DOI:** 10.3390/jof12010079

**Published:** 2026-01-22

**Authors:** Marta Maria Barone, Marco Moizio, Ravish Choudhary, Chiara D’Errico, Vojislav Trkulja, Livio Torta, Salvatore Davino, Slavica Matić

**Affiliations:** 1 Department of Agricultural, Food and Forest Sciences (SAAF), University of Palermo, Viale delle Scienze, 90128 Palermo, Italy; martamaria.barone@unipa.it (M.M.B.); livio.torta@unipa.it (L.T.); salvatore.davino@unipa.it (S.D.); 2 SAGEA Centro di Saggio s.r.l., Via San Sudario 15, 12050 Castagnito, Italy; marco.moizio@sagea.com; 3 Division of Seed Science and Technology, ICAR-Indian Agricultural Research Institute, New Delhi 110012, India; ravianu1110@gmail.com; 4 Institute for Sustainable Plant Protection (IPSP), National Research Council of Italy (CNR), Strada delle Cacce 73, 10135 Torino, Italy; chiara.derrico@cnr.it; 5 Agricultural Institute of Republic of Srpska, Knjaza Milosa 17, 78000 Banja Luka, Bosnia and Herzegovina; vtrkulja@blic.net; 6 Faculty of Agriculture, University of Banja Luka, Bulevar Vojvode Petra Bojovića 1A, 78000 Banja Luka, Bosnia and Herzegovina; 7 Academy of Sciences and Arts of the Republic of Srpska, Bana Lazarevića 1, 78000 Banja Luka, Bosnia and Herzegovina

**Keywords:** *Erysiphe corylacearum*, hazelnut, LAMP assay, powdery mildew, field diagnostics

## Abstract

*Erysiphe corylacearum*, the causal agent of powdery mildew in hazelnut (*Corylus avellana* L.), has become an emerging pathogen of concern in Italian hazelnut production requiring rapid and accurate detection to support timely disease management and phytosanitary measures. We developed and validated a field-deployable loop-mediated isothermal amplification (LAMP) assay for the specific detection of *E. corylacearum* and evaluated three primer sets targeting the Internal Transcribed Spacer (ITS) region, RNA polymerase II second largest subunit *(rpb2*), and glutamine synthetase (*GS*) genes; the *GS*-targeting Ecg set showed the highest sensitivity and specificity. The assay was shown to be sensitive down to 200 fg of fungal DNA, efficiently detected *E. corylacearum* from diluted crude leaf extracts, and produced results within half an hour, allowing the detection of latent infections before visible symptoms emerged. On-site validation with a portable LAMP instrument showed the assay’s suitability for field-deployable diagnosis and early-warning applications in hazelnut orchards.

## 1. Introduction

*Erysiphe corylacearum* [1] is an obligate biotrophic fungal pathogen that causes powdery mildew disease in hazelnut (*Corylus avellana* L.) and other *Corylaceae* species. This disease has emerged as a significant threat in several hazelnut-growing regions [2,3], resulting in considerable economic losses due to reduced nut yield and quality. *E. corylacearum* is causing increasing damage to hazelnut cultivation throughout the European Union, where it is considered an emerging pathogen. Originally found only in Asia and America on other hazelnut species (Asian, Japanese, and beaked hazelnut), it was able to expand its host range and spread in less than a decade to hazelnut trees from Turkey [4], Iran [5] and Israel [6] into Eastern, Central and Southern Europe, including Northern Italy [7,8]. Moreover, there is a risk of further expansion into new geographic regions. Early and accurate detection of *E. corylacearum* is essential for timely disease management and the implementation of effective phytosanitary measures, especially since only a limited number of fungicides are available against this pathogen. The implementation of integrated pest management (IPM) measures, based on early monitoring and targeted interventions, is essential. This requires the use of diagnostic methods that are rapid, sensitive, tolerant to sample inhibitors, and operable on crude extracts. In this context, the development and validation of a targeted LAMP assay for *E. corylacearum* would provide a practical diagnostic tool to support timely IPM decision-making.

Traditional diagnostic methods, such as visual inspections and microscopic examinations, are often insufficient due to the cryptic nature of early infections and the morphological similarity to other powdery mildew species. A molecular approach based on PCR, developed by Cunnington [9] and targeting the Internal Transcribed Spacer (ITS) region of ribosomal DNA, demonstrated effectiveness and high specificity in linking anamorphic Erysiphales to their corresponding teleomorphs across 12 tested genera, even in samples containing contaminant fungi.

For species identification, various authors have performed Multilocus Sequence Typing (MLST) targeting different genes. Takamatsu et al. [10] analyzed the ITS region, and subsequently Bradshaw et al. [11] explored four additional genes: RNA polymerase II second largest subunit (*rpb2*), calmodulin (*CaM*), glyceraldehyde 3-phosphate dehydrogenase (*GAPDH*), and glutamine synthetase (*GS*). These genes were later used by Matić et al. [8] for further molecular diversity investigations.

Although PCR-based and MLST methods are sensitive and specific, they are limited by high costs, long running times, and the requirement for laboratory facilities and trained personnel. For these reasons, there is a growing need to develop alternative techniques, among which loop-mediated isothermal amplification (LAMP) has gained attention as a rapid, cost-effective, and field-deployable method for pathogen identification [12,13]. LAMP offers several advantages over conventional PCR [14], including operation at a constant temperature, reduced time to result, and tolerance to sample inhibitors, making it suitable for field applications [15]. However, the success of LAMP assays largely depends on the design of highly specific primers targeting unique genomic regions of the pathogen of interest, as well as the optimization of reaction conditions.

Since no LAMP-based molecular assay has been developed to date for *E. corylacearum*, our objective was to develop and validate a novel LAMP test for the specific detection of this fungus. Furthermore, we aimed to set up a crude plant extract procedure suitable for in-field use, thereby avoiding laborious DNA extraction with commercial kits.

## 2. Materials and Methods

### 2.1. DNA Extraction

Ten mg of epiphytic fungal structures (hyphae, conidiophores, and conidia) scraped from symptomatic leaves of a naturally infected hazelnut plant (from which the *E. corylacearum* isolate 8_PdIT was previously identified) [7] were collected in June 2024. This fungal material was used for DNA extraction as the positive control. In addition, 100 mg of leaves from healthy hazelnut trees cultivated at IPSP-CNR, Turin, uninfected with *E. corylacearum*, were also collected and used for DNA extraction as the healthy negative control. DNA was extracted using the GenUP Plant DNA Kit (Biotechrabbit, Berlin, Germany) in three biological replicates. DNA quality and concentration were assessed spectrophotometrically (NanoDrop 2000, Thermo Scientific, Waltham, MA, USA).

### 2.2. Primer Design for LAMP Assay

Three LAMP primer sets were designed targeting different loci: *rpb2* (Ecr1), ITS (Eci), and *GS* (Ecg), based on the *E. corylacearum* sequences of the 8_PdIT isolate (OR126177, OQ917090, and OR126213), using PrimerExplorer v.5 software (Eiken Chemical Corporation, Tokyo, Japan).

The first set, designed within the *rpb2* gene, contained 4 primers lacking the loop (LF and LB) primers. The second set, designed within the ITS region, consisted of all six common LAMP primers, while the third one, designed within the *GS* gene, included five primers (lacking the loop LF primer). Primer specificity was evaluated using the Nucleotide BLAST algorithm (https://www.ncbi.nlm.nih.gov, accessed on 1 December 2025) by aligning the primer sequences against other hazelnut-associated powdery mildew and pathogenic fungi to exclude potential cross-reactivity.

### 2.3. Optimization of the LAMP Assay

LAMP assay was performed in a reaction mixture consisting of 6.25 μL 2× SuperScript IV RT-LAMP Master Mix (Invitrogen, Thermo Fisher Scientific Baltics UAB, Vilnius, Lithuania), 0.5 μL of each internal primer (FIP and BIP), 0.05 μL of each external primer (F3 and B3), and 0.25 μL of each loop primer (when included), 1.25 μL SYTO Green Fluorescent Nucleic Acid Stain (Invitrogen), 1.5 μL of DNA (35 ng in total), and water to a final volume of 12.5 μL. For the *rpb2* primer set, no loop primers were used; for the ITS primer set, both loop primers were included; and for the *GS* primer set, only the LB loop primer was added. All primers were added from stock solutions at a concentration of 50 μM.

LAMP assay was performed for 60 min, using three different annealing temperatures (60 °C, 63 °C and 65 °C), followed by enzyme inactivation at 80 °C for 5 min. Melting curves were generated from 65° C to 95 °C at a ramp rate of 0.1 °C with readings performed every 15 s. The assay was carried out on Rotor-Gene Q (QIAGEN Benelux B.V., Venlo, The Netherlands). Two technical replicates were used for each sample (positive control, negative control, and non-template control; NTC).

### 2.4. Sensitivity and Specificity Evaluation

To assess sensitivity, a ten-fold serial dilution of DNA (20 ng/μL) of *E. corylacearum* positive control and two naturally infected hazelnut isolates (1.1_PdIT and 7.1_PdIT) was prepared, ranging from 10^−1^ to 10^−6^. DNA from the 1.1_PdIT and 7.1_PdIT isolates was obtained by collecting epiphytic fungal structures as described in Section 2.1. Dilutions were prepared in nuclease-free water, and each concentration was tested in three technical replicates.

To test analytical specificity, the LAMP assay was assessed using DNA extracted from healthy hazelnut tissue and from isolates of other powdery mildew species from ha-zelnut, grapevine, apple and peach, originated from Italy (reported in Table S1).

### 2.5. Comparison of LAMP with PCR Specific Assay

Finally, the LAMP assay was compared with another molecular technique, the *E. corylacearum*-specific PCR [16] for further confirmation of the suitability of the developed assay. Repeated-measures one-way ANOVA has been used to compare the two techniques.

### 2.6. Development of a Rapid Crude Extract Method for LAMP

To make the optimized LAMP method suitable for field application, different sample preparations were first tested in the laboratory. To evaluate the best protocol for preparing crude plant extract, various extraction buffers and homogenization procedures were tested. One hundred mg of the plant leaves from the positive and negative control trees was placed in a 2 mL sterile tube with a steel bead (2 mm diameter) containing 1 mL of: (a) ELISA extraction buffer (1× phosphate-buffered saline, pH 7.2; Tween 20 0.05%, polyvinylpyrrolidone 2%), (b) lysis buffer LS (Biotechrabbit, Berlin, Germany) and (c) TET buffer (20 mM Tris-HCl pH 8; 20 mM EDTA, 1% Triton X-100) [17]. The plant material was homogenized for 2–3 min either by vortexing or manual vigorous shaking, and the resulting sap was added directly to the LAMP mixture or after serial dilutions (1:10, 1:100, 1:1000). The most effective extraction and homogenization method was selected in the laboratory and used for subsequent LAMP assays.

### 2.7. On-Site LAMP Assay

During spring-summer 2024 (June–August), ten hazelnut orchards of the ‘Tonda Gentile delle Langhe’ cultivar were visited in Asti, Turin, and Cuneo provinces, within the Piedmont region. From each orchard, 10 symptomatic leaves and 10 asymptomatic leaves were collected from previously identified trees infected with *E. corylacearum* [8] during both the latent phase (before symptom appearance) and the symptomatic phase (when powdery mildew was visible on the upper leaf surface).

Samples were processed in the field using the TET-based crude-extract protocol and manual homogenization, as described in Section 2.6. Crude plant extracts were diluted 1:10 in nuclease-free water, and 1.5 µL of the diluted crude plant extract was directly loaded into LAMP reactions prepared on-site. Reactions were run on a portable thermocycler (Hyris bCUBE2, Hyris Ltd., London, United Kingdom) at 60 °C with real-time fluorescence monitoring and subsequent melting-curve verification. Each biological sample was assayed in two technical replicates. DNA from epiphytic *E. corylacearum* structures, healthy hazelnut DNA, and sterile water were included as the positive control, negative control, and NTC, respectively.

## 3. Results

### 3.1. Design of LAMP Primers

**Figure 1:**
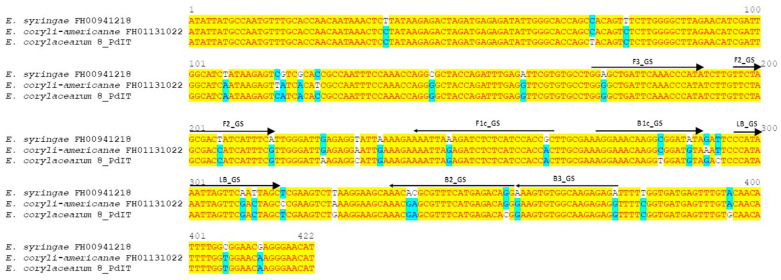
Schematic representation of LAMP primers designed in *Erysiphe corylacearum* glutamine synthetase (*GS*) gene for specific fungal detection. All primers are indicated by arrows above the sequence alignment from *Erysiphe* spp. isolates that infect hazelnut available in GenBank, including the 8_PdIT reference isolate from Italy (Matić et al., 2024 [8]). F3 and B3 are external primers, and LB is a loop primer. F1c and F2 portions form the FIP primer, and B1c and B2 fragments form the BIP primer.

Three sets of specific primers were designed in the *rpb2* gene, the *GS* gene and the ITS region to develop a real-time LAMP assay for the specific detection of *E. corylacearum*. The primer sequences and positions are reported in Table 1 and Figure 1, respectively. No cross-reactions with other organisms were found during the in silico analysis using the Nucleotide-BLAST algorithm (BLASTn). Furthermore, hybridization analysis carried out with the Vector NTI 11.5 software (Invitrogen, Carlsbad, CA, USA) ruled out any matches with other powdery mildew fungal species.

### 3.2. Optimization of the LAMP Assay

**Figure 2:**
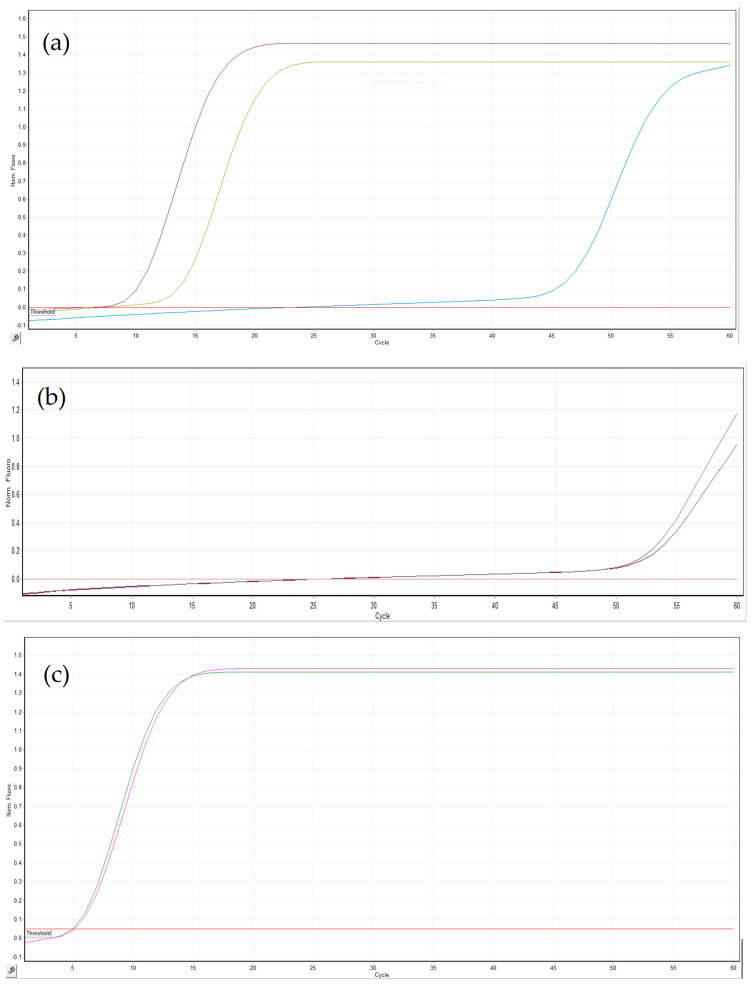
Amplification curves of the LAMP reaction at 60 °C of two undiluted DNA samples extracted from epiphytic *E. corylacearum* structures (hyphae, conidiophores, and conidia) collected from hazelnut leaves using (**a**) Eci set, (**b**) Ecr1 set, and (**c**) Ecg set of primers. Signals from healthy controls are shown in red and blue in (**a**), and in red in (**b**,**c**).

The three LAMP primer sets reported in Table 1 were tested on DNA extracted from epiphytic fungal structures (hyphae, conidiophores, and conidia) at a concentration of 20 ng/uL and on total DNA extracted from a healthy plant (Figure 2). The Eci set showed unspecific reactions with the healthy control and lower reproducibility between technical replicates, while the Ecr1 set exhibited slower amplification (time to positive greater than 45 min). On the other hand, the Ecg set, targeting the *GS* gene, produced the most reliable results in terms of sensitivity, specificity, and reproducibility. The higher efficiency of the Ecg primer set is most likely related to more favorable primer-binding features of the *GS* gene and its higher level of conservation. Therefore, the Ecg set was selected as the best-performing LAMP primer set for further experiments.

To further optimize the Ecg primer set, LAMP reactions were compared at three isothermal temperatures: 60 °C, 63 °C, and 65 °C. The optimal amplification occurred at 60 °C, with early signals and high reproducibility of technical replicates. At 63 °C and 65 °C, amplification was still detectable but occurred later, indicating reduced assay efficiency at higher temperatures (Table 2 and Figure S1).

**Figure 3:**
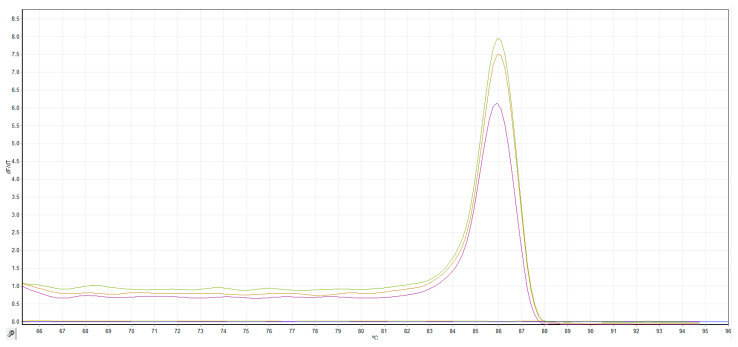
Melting curves of *E. corylacearum* from two isolates and the positive control, using DNA obtained from epiphytic fungal structures of naturally infected hazelnut plants.

Melting curve analysis of the optimized LAMP revealed a single sharp peak (86 °C) (Figure 3), confirming the presence of a specific product and the absence of non-specific amplification or primer-dimer formation. Negative controls, including healthy hazelnut DNA and no-template reactions, consistently showed no amplification. Based on the obtained results, all successive LAMP assays were performed at annealing temperature of 60 °C.

### 3.3. Sensitivity, Specificity and Repeatability of the LAMP Assay

In order to test sensitivity, a ten-fold serial dilution series (10^−1^ to 10^−6^) was prepared, starting from 20 ng/uL of *E. corylacearum* DNA. Dilutions were prepared in nuclease-free water, and each concentration was tested in three technical replicates. Within the tested dilution range, the optimized LAMP assay demonstrated a detection limit of up to 10^−5^ dilution of purified DNA in both the positive control and naturally infected hazelnut isolates (1.1_PdIT and 7.1_PdIT). Thus, LAMP reproducibly detected *E. corylacearum* down to 200 fg, establishing the assay’s detection limit under the conditions described (Table 3 and Figure S2). Furthermore, the best dilution for DNA extracts was 10^−1^, which showed the earliest amplification curves, along with undiluted DNA.

On the other hand, to test the analytical specificity, the LAMP assay was assessed using DNA extracted from healthy hazelnut tissue and from other fruit-tree powdery mildew species reported in Table S1 to confirm the absence of cross-reactivity. The LAMP assay showed no cross-reactivity with DNA from healthy hazelnut tissues or other powdery mildew species.

Repeatability was evaluated by repeating the assay on three different isolates of DNA under the best-performing conditions at a concentration of 2 ng/µL. Each sample was tested in two technical replicates. Results showed that the LAMP assay was repeatable, without important differences in detection time across different runs (±1 Tp).

### 3.4. Comparison of LAMP with a Specific PCR Assay

**Figure 4:**
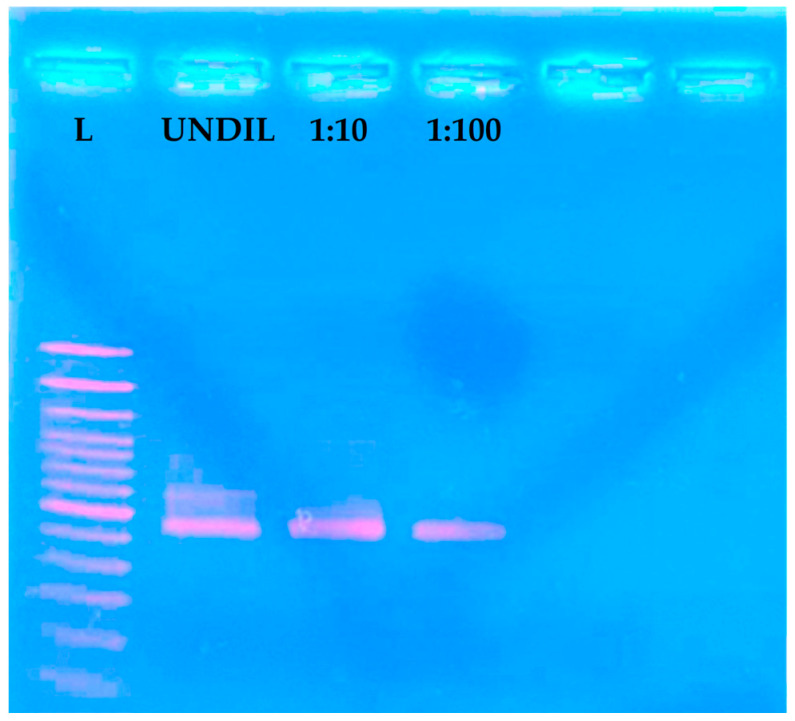
*E. corylacearum*-specific end-point PCR assay performed on a serial dilution of DNA extracted from epiphytic fungal structures collected from the surface of infected hazelnut leaves. From left to right: L = DNA Ladder (Thermo Scientific^TM^ GeneRuler 100 bp, Fisher Scientific, Loughborough, UK), UNDIL = Undiluted DNA, 1:10 = 10^−1^ dilution, and 1:100 = 10^−2^ dilution.

Comparison of the LAMP assay with the *E. corylacearum*-specific PCR showed a superior detection limit of LAMP technique using the DNA extracts from epiphytic fungal structures compared to the specific PCR (200 fg with LAMP versus 200 pg with PCR) (Figure 4) and a time saving of approximately 3 h.

### 3.5. Comparison of Different Crude Extract Methods

**Figure 5:**
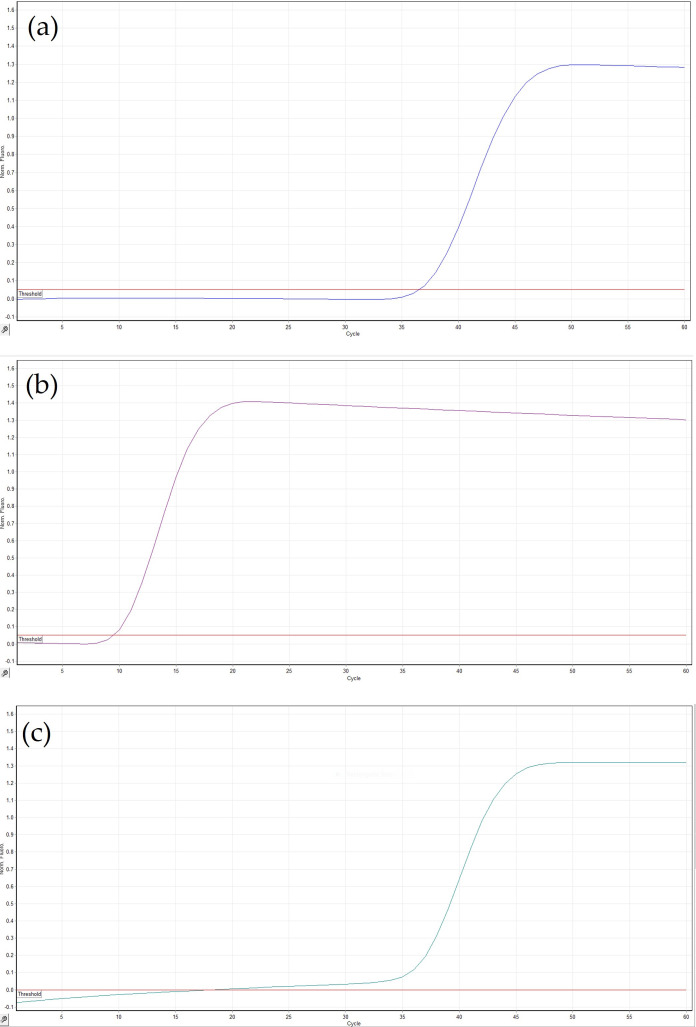
LAMP detection of *E. corylacearum* in crude plant extracts diluted 10^−1^ by using (**a**) lysis, (**b**) TET and (**c**) ELISA buffers.

During the preparation of crude plant extracts from hazelnut leaves, manual shaking during extraction gave more homogeneous results compared to vortexing. Assessing three different extraction buffers, TET buffer outperformed both the ELISA extraction buffer and the LS lysis buffer, yielding faster and more robust LAMP amplification signals and higher reproducibility. Crude extracts prepared with TET produced clear positive amplifications at dilutions down to 1:1000, whereas the undiluted extract did not yield amplifiable results. TET-treated samples consistently yielded more uniform Tp values across both vortex and manual vigorous shaking homogenization methods. By contrast, samples prepared with the ELISA buffer exhibited inhibition of the LAMP reaction until a 1:100 dilution, while samples prepared with the LS lysis buffer yielded amplification at a 1:10 dilution but showed consistently delayed time to positivity compared with TET-extracted samples (Figure 5). Based on these findings, the TET extraction protocol and manual shaking method were selected for subsequent laboratory LAMP assays and for further development of the field-deployable procedure.

### 3.6. LAMP Assay with DNA Extracted from Field Samples

Ten samples collected from symptomatic and asymptomatic hazelnut leaves in Cuneo province were processed simultaneously using both purified DNA and TET-based crude extract protocols (Table 4). DNA extracts showed rapid and consistent amplification, with Tp values ranging from 4.05 to 12.6 min. Crude extracts produced slightly delayed but comparable amplification, with Tp values between 5.84 and 13.97 min. Statistical analysis employing repeated-measures ANOVA and Tukey’s HSD test (Table 4) showed that for several isolates, amplification from crude extracts was slightly delayed compared to purified DNA (*p* ≤ 0.05), whereas for others there was no significant variation (*p* > 0.05). Importantly, TET-based crude extract amplification was obtained in both symptomatic and asymptomatic leaf samples, demonstrating the ability of the assay to detect latent infections. In all positive cases, detection occurred in less than 30 min. Based on these results, simplified TET crude extracts at a 10^−1^ dilution were prepared in all in-field assays.

### 3.7. On-Site LAMP Assay with Crude Plant Extracts

**Figure 6:**
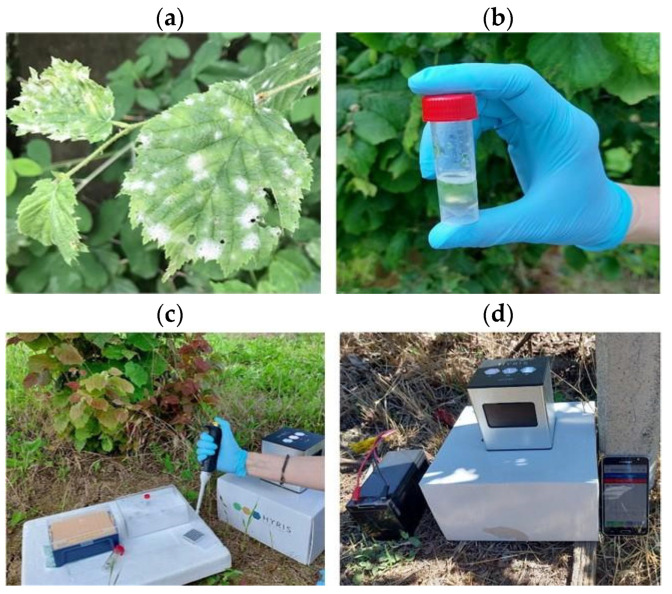
In-field detection of *Erysiphe corylacearum* in hazelnut using real-time LAMP assay: (**a**) symptomatic hazelnut leaves; (**b**) preparation of plant crude extracts; (**c**) preparation of the LAMP reaction mixture; (**d**) running of the LAMP assay in hazelnut orchard by bCube2 instrument and visualization of the results on the smartphone.

To evaluate assay performance under point-of-care conditions, 20 non-symptomatic and 20 symptomatic hazelnut leaf samples were collected during the June-August period of 2024 from 10 orchards in 3 Piedmont areas (Turin, Asti and Cuneo provinces). Thus, 100 mg of non-symptomatic and symptomatic hazelnut leaf tissues were homogenized on-site using TET buffer as described in Section 2.6 and diluted 1:10 in nuclease-free water. LAMP reaction mix was prepared as described in Section 2.3, and reactions were run directly in the field at 60 °C for 60 min on a portable thermocycler (Hyris bCUBE2^®^, Hyris Ltd., London, United Kingdom) (Figure 6). This on-site LAMP assay detects *E. corylacearum*, enabling rapid, field-deployable diagnosis without laboratory DNA purification.

Symptomatic samples typically yielded Tp values between ~4 and 9 min, while asymptomatic/latent samples required longer Tp (≈10–40 min). These results confirmed that the optimized crude-extract LAMP workflow was suitable for rapid in-field diagnosis of *E. corylacearum* without prior nucleic acid purification.

## 4. Discussion

The LAMP technique is one of the most widely used innovative methods for the detection of plant pathogens. This is possible due to its rapidity, cost-effectiveness, specificity, and reproducibility, as well as its suitability for field deployment without reliance on time-consuming and costly DNA extraction kits. To date, the LAMP assay has been employed primarily for the detection of powdery mildew pathogen of grapevine [18,19,20]. However, it has not yet been developed for the detection of powdery mildew causal agents in other perennial fruit crops, including the emerging powdery mildew of hazelnut.

In this study, we successfully developed a sensitive LAMP assay for the specific detection of *E. corylacearum.* The Ecg primer set, designed specifically for *E. corylacearum,* demonstrated superior sensitivity compared to the Ecr1 and Eci primer sets, and was suitable for field-deployable diagnosis. The developed LAMP assay also overcame the limitations of traditional laboratory-based detection methods, and when compared to specific-PCR developed by Kalmár et al. [16], it showed up to 100-fold higher sensitivity, producing results within approximately 3 h. Thiessen et al. [20] developed a quantitative LAMP assay for *Erysiphe necator* and demonstrated a theoretical limit of detection approaching a single conidium under controlled laboratory conditions. In that study, analytical sensitivity was expressed in terms of spore number rather than as mass or copy number of DNA. Additionally, because the assay exhibited reduced performance at very low inoculum densities in field deployments, it could not be directly compared with the developed LAMP assay. In our study, analytical sensitivity was determined using quantified DNA extracted from epiphytic fungal structures (hyphae, conidiophores, and conidia), revealing a detection threshold of 200 fg. The assay also maintained high amplification efficiency even when crude plant extracts obtained with TET buffer were used. Similar findings were documented by Vettraino et al. [21], who reported limits of detection of 0.128 pg µL^−1^ for qPCR and P-LAMP, and 0.64 pg µL^−1^ for V-LAMP in assays targeting *Gnomoniopsis smithogilvyi*. Both studies confirmed the high-performance of the LAMP system, which typically operates within the low-picogram to femtogram DNA range.

Within this methodological landscape, our LAMP assay for *E. corylacearum* demonstrates analytical sensitivity fully consistent with those of previously published field-deployable LAMP protocols. The assay reliably detected pathogen at dilutions down to 10^−5^ from DNA and 10^−3^ from crude extracts, while consistently yielding rapid Tp values (4–13 min in symptomatic tissues). Notably, the assay preserved its diagnostic performance in simplified TET-based preparations, eliminating the need for laboratory-grade DNA purification and enabling robust amplification under field conditions. High specificity, absence of cross-reactivity with other hazelnut-related powdery mildew species, and repeatability further reinforce the operational reliability of the method.

Overall, our results position this assay as one of the highly sensitive and field-compatible LAMP tools currently available for fungal plant pathogens in nut trees, combining high analytical performance with procedural simplicity and suitability for large-scale phytosanitary surveillance [21,22,23]. However, due to the limited number of isolates used in this study, future studies need to evaluate additional isolates to further confirm the sensitivity and specificity features of the developed LAMP assay.

Performing molecular analyses in the field, LAMP assays using crude plant extracts confer operational and economic advantages for rapid diagnostics. By avoiding laborious DNA extraction and purification steps, sample preparation is simplified, turnaround time is reduced, and reliance on specialized laboratory infrastructure is minimized; LAMP’s single-temperature operation and tolerance to diluted crude matrices further eliminate the need for extraction columns and other consumables that substantially increase per-sample costs [17,24,25]. Specifically, workflows that use kit-based DNA extraction and conventional PCR test generally require over 4 h from sample to result, whereas crude-extract LAMP test provides results in less than 1 h, offering a substantial time saving. Based on our cost model, which incorporates reagent and consumable prices, basic field equipment, operator time and an amortized share of instrument cost distributed over expected throughput, the estimated per-sample cost for a crude-extract LAMP assay is approximately €1.15, compared with €5.5–6.0 per sample for workflows that include kit-based DNA extraction and end-point PCR; these estimates are indicative and will vary with local pricing, operator wages and scale of analysis. Consequently, crude-extract LAMP offers a lower-cost alternative for point-of-need pathogen detection, reducing infrastructure and consumable requirements while increasing accessibility and throughput relative to conventional PCR workflows. The portability and rapid execution of the LAMP workflow make it particularly well suited for integration into early-warning systems and IPM programmes: on-site diagnosis enables timely, site-specific interventions that can reduce fungicide applications and contribute to resistance management.

The reaction temperature was shown to be a critical factor, with 60 °C identified as the optimal condition. Amplification was still achievable at 63 °C and 65 °C, though with delayed signal onset and high intra-sample variability, confirming the temperature-dependence typical of LAMP assays. These results are in line with findings from similar studies, where optimization of isothermal conditions significantly influenced detection efficiency [26,27,28,29].

Another major advantage of the assay is its compatibility with diluted crude plant extract. Utilization of the TET buffer and manual homogenization provided consistent amplification, relative to alternative extraction buffers evaluated, resulted in superior and more reproducible amplification curve profiles, eliminating the need for laboratory-grade DNA extraction. Moreover, the TET buffer should be considered in the future as a versatile buffer for preparing crude plant extracts, as it is effective not only for powdery mildew detection, as shown here, but also for the identification of other plant pathogens such as bacteria [25], phytoplasmas [24], and viruses [17,28,30].

The developed LAMP assay has several intrinsic limitations that merit explicit consideration. The assay yields qualitative or semi-quantitative outputs: although time-to-positive (Tp) values may correlate with template abundance, LAMP is not inherently quantitative without rigorous calibration curves, validated controls and normalization strategies. Secondly, despite comprehensive in silico and empirical specificity testing against available reference sequences and related taxa, diagnostic performance may be compromised by the emergence or identification of novel, closely related strains bearing mutations within primer binding sites; therefore, ongoing sequence surveillance and periodic primer revalidation are necessary to maintain assay accuracy. Finally, field deployment introduces additional sources of variability, sample heterogeneity, variable inhibitor loads, operator skills and environmental conditions, that can affect sensitivity and reproducibility; these risks should be mitigated through the inclusion of internal amplification controls, standardized sampling and extraction protocols, operator training and, where necessary, confirmatory laboratory testing.

Collectively, these findings validate the developed LAMP assay as a powerful tool for the early and accurate detection of *E. corylacearum*, with strong potential for implementation in routine plant health monitoring and disease management strategies.

## 5. Conclusions

This study reports the development and validation of a loop-mediated isothermal amplification (LAMP) assay specifically designed for the detection of *E. corylacearum*, the emerging causal agent of powdery mildew in hazelnut. Among the tested primer sets, the Ecg set targeting the glutamine synthetase (*GS*) gene demonstrated the highest diagnostic efficiency, providing rapid, sensitive, and specific amplification. The assay was effective not only with purified DNA but also with diluted crude plant extracts, particularly when using the TET buffer. This eliminates the need for DNA purification procedures, making the protocol ideal for in-field analysis.

The assay’s ability to detect the pathogen within 40 min of reaction initiation, even from unprocessed samples, positions it as a practical tool for early intervention and large-scale monitoring in hazelnut orchards. Moreover, a rapid pathogen detection offers a clear advantage over traditional PCR methods, particularly during early asymptomatic stages of infection. Given its reliability and operational simplicity, the assay offers a valuable contribution to phytopathological diagnostics and could be integrated into surveillance programs to help mitigate the spread and impact of powdery mildew caused by *E. corylacearum* in hazelnut production systems.

Future studies will be dedicated to developing a multiplex LAMP assay capable of simultaneously identifying *Phyllactinia guttata* and *Erysiphe corylacearum,* the causal agents of common and emerging powdery mildew on hazelnut, respectively. This approach would enable the assessment of mixed infections and the epidemiological monitoring of both pathogen populations, particularly in the context of climate change [31,32,33].

## Figures and Tables

**Table 1:** Three LAMP primer sets targeting different loci: *rpb2* (Ecr1), *GS* (Ecg), and ITS (Eci).

Primer Sets	Name	Sequence (5′–3′)	Gene	Primer Position	Final Concentration in LAMP Reaction
1	F3_Ecr1	AGACAGTTCAAAATTTACGCC	*rpb2*	350–370	0.2 µM
	B3_Ecr1	TGATCGTCCTCAAGCCTA		537–554	0.2 µM
	FIP_Ecr1	ACACGACCTGCATCTGTAAATATTT-GGTCTCATTTGATCTCTCACG		46	2.0 µM
	BIP_Ecr1	TGTTATTGATAATGACCCTGAGAGC-CGAATATGCTCTTTATTAAGAACC		49	2.0 µM
2	F3_Ecg	GGGCTGATTCAAACCCATA	*GS*	172–190	0.2 µM
	B3_Ecg	CCTCTCTTGCCACACTTC		358–375	0.2 µM
	FIP_Ecg	AGTGGTGGATGAGAGATCTCTAATT-TTCTAGCGACCATCATTTCG		45	2.0 µM
	BIP_Ecg	AGGAAACAAGGTGGATGTAGACT-GTCTCATGAAACGCTCGT		41	2.0 µM
	LB_Ecg	CCCATAAATTAGTTCGACTAGCTCG		295–319	1.0 µM
3	F3_Eci	TGTTCGAGCGTCATAACACC	ITS	440–459	0.2 µM
	B3_Eci	GGTCAACCTGTGATCCATGT		618–637	0.2 µM
	FIP_Eci	CTGTCTTTAAGGGCCGCCGC-CCTCCAGCTGCCTTTGTG		38	2.0 µM
	BIP_Eci	GCGTGGGCTCTACGCGTAGTA-TTTTGGCAAGCCACCGTC		39	2.0 µM
	LF_Eci	CCCCAACACCGCAACCA		479–495	1.0 µM
	LB_Eci	ACTTGCTTCTCGCGACAGA		559–577	1.0 µM

**Table 2:** Detection of *Erysiphe corylacearum* from two isolates and the positive control obtained from DNA of epiphytic fungal structures (hyphae, conidiophores, and conidia) scraped from the surface of naturally infected hazelnut leaves at annealing temperatures of 60 °C, 63 °C, and 65 °C.

**Isolate**	Time to positive (Tp) in minutes	**Isothermal Temperature**		
		**60 °C**	**63 °C**	**65 °C**
Positive control		5	32	34
8.2_PdIT		6	37	35
8.3_PdIT		6.5	40	37

**Table 3:** Detection of *E. corylacearum* in two naturally infected hazelnut isolates and positive control at different dilutions.

Isolate	Sample Type	Tp * (Min) ± SD ** at Different Dilutions						
		20 ng/μL	10^−1^	10^−2^	10^−3^	10^−4^	10^−5^	10^−6^
1.1_PdIT	DNA from epiphytic fungal structures	4.05 ± 0.34	5.04 ± 0.8	36.24 ± 0.80	37.37 ± 1.20	45.43 ± 2.82	47.43 ± 1.26	ND ***
7.1_PdIT		4.13 ± 0.23	4.9 ± 1.09	36.57 ± 2.32	41.06 ± 0.82	46.99 ± 1.47	49.87 ± 0.18	ND
Positive control		4.7 ± 0.15	5.98 ± 0.05	32.32 ± 1.14	36.11 ± 4.22	39.74 ± 0.91	49.41 ± 3.42	ND

* Tp = time to positive; ** SD = standard deviation; *** ND = not determined.

**Table 4:** LAMP detection of *E. corylacearum* in the laboratory using DNA extracts and crude plant extracts diluted 1:10. Statistical significance analyzed using repeated-measures one-way ANOVA. Different letters showed significant differences according to Tukey’s HSD test (*p*-value ≤ 0.05).

Isolate	Tp * (Min) ± SD **		
	DNA	Crude Plant Extract	*p*-Value
1_PdIT.1 (S) ***	4.05 ± 0.34 ^a^	5.92 ± 1.37 ^a^	0.0878 n.s. ****
7_PdIT.1 (S)	4.13 ± 0.23 ^a^	7.43 ± 0.24 ^b^	0.0002
8_PdIT.1 (S)	4.7 ± 0.15 ^a^	8.05 ± 2.10 ^a^	0.1060 n.s.
5_PdIT.1 (AS) *****	9.74 ± 0.06 ^a^	12.60 ± 1.50 ^a^	0.0777 n.s.
49_PdIT (S)	8.63 ± 0.01 ^a^	11.33 ± 0.50 ^b^	0.0002
50_PdIT (S)	9.52 ± 0.17 ^a^	13.97 ± 0.29 ^b^	0.0002
51_PdIT (AS)	10.03 ± 0.39 ^a^	12.87 ± 0.37 ^b^	<0.0001
52_PdIT (AS)	10.61 ± 0.92 ^a^	5.84 ± 0.68 ^b^	0.0008
53_PdIT (S)	7.94 ± 0.20 ^a^	8.02 ± 0.09 ^a^	0.6893 n.s.
54_PdIT (AS)	12.6 ± 1.07 ^b^	10.80 ± 0.80 ^a^	0.0074

* Tp = time to positive; ** SD = standard deviation; *** S = symptomatic sample; **** n.s. = non significant; ***** AS = asymptomatic sample.

## Data Availability

The original contributions presented in this study are included in the article/Supplementary Material. Further inquiries can be directed to the corresponding author.

## Supplementary Materials

The following supporting information can be downloaded at: https://www.mdpi.com/article/10.3390/jof12010079/s1. Table S1: Powdery mildew species used to test the cross reactivity of the LAMP assay; Figure S1: Amplification curves of the LAMP reaction for three biological replicates of DNA extracted from epiphytic Erysiphe corylacearum structures (hyphae, conidiophores, and conidia) infecting hazelnut leaves, at different annealing temperatures. (a) 60 °C, (b) 63 °C and (c) 65 °C. Figure S2: Amplification curves of the LAMP reaction at 60 °C of a positive sample (*E. corylacearum* DNA) diluted from 10⁰ to 10^−^⁵ (1 = undiluted DNA, 2 = diluted 10^−1^, 3 = diluted 10^−2^, 4 = diluted 10^−3^, 5 = diluted 10^−4^, 6 = diluted 10^−5^). Figure S3. Amplification curves of the LAMP reaction at 60 °C of a positive sample of crude plant extract, obtained using TET buffer, diluted from 10⁰ to 10^−3 ^(7 = undiluted crude plant extract, 8 = diluted 10^−1^, 9 = diluted 10^−2^, 10 = diluted 10^−3^).
