# Supplementary material for: Development of a Field-Deployable Loop-Mediated Isothermal Amplification Assay for the Rapid Detection of Erysiphe corylacearum in Hazelnut
Source: J Fungi (Basel). 2026 Jan 22;12(1):79. doi: 10.3390/jof12010079 (PMC12842720; doi:10.3390/jof12010079)

Supplementary Materials

Journal of Fungi

Article

Development of a Field-Deployable Loop-Mediated Isothermal Amplification Assay for the Rapid Detection of *Erysiphe corylacearum* in Hazelnut

Marta Maria Barone, Marco Moizio, Ravish Choudhary, Chiara D'Errico, Vojislav Trkulja, Livio Torta, Salvatore Davino, and Slavica Matić

Table S1. Powdery mildew species used to test the cross reactivity of the LAMP assay.

Species	Isolate	Host	Geographical origin	Source
Erysiphe necator	*PDEN*	Grapevine	Italy	IPSP-CNR, Torino
Podosphaera pannosa	*PS0*	Peach	Italy	SAAF, University of Palermo
Podosphaera leucotricha	*IPL*	Apple	Italy	IPSP-CNR, Torino
Phyllactinia guttata	*PDPG1*	Hazelnut	Italy	IPSP-CNR, Torino

Figure S1 Amplification curves of the LAMP reaction for three biological replicates of DNA extracted from epiphytic *Erysiphe corylacearum* structures (hyphae, conidiophores, and conidia) infecting hazelnut leaves, at different annealing temperatures. a) 60 °C, b) 63 °C and c) 65 °C.

(a)

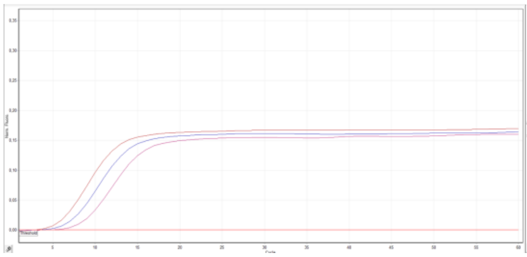

(b)

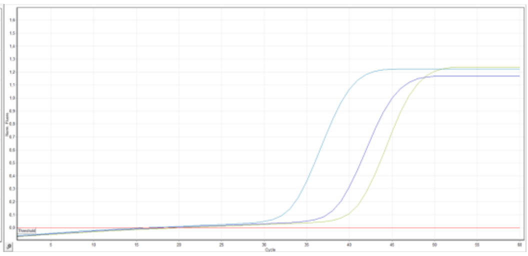

(c)

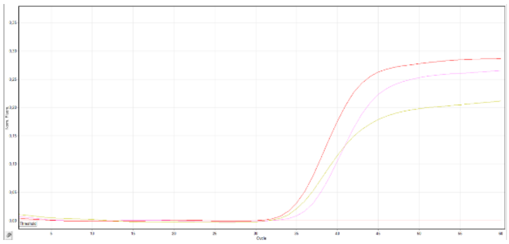

Figure S2. Amplification curves of the LAMP reaction at 60 °C of a positive sample (*E. corylacearum* DNA) diluted from 10^{0} to 10^{-5} (1 = undiluted DNA, 2 = diluted 10^{-1}, 3 = diluted 10^{-2}, 4 = diluted 10^{-3}, 5 = diluted 10^{-4}, 6 = diluted 10^{-5}).

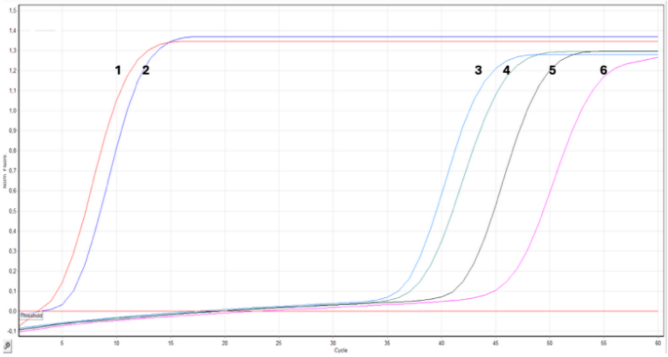

Figure S3. Amplification curves of the LAMP reaction at 60 °C of a positive sample of crude plant extract, obtained using TET buffer, diluted from 10^0 to 10^{-3} (7 = undiluted crude plant extract, 8 = diluted 10^{-1}, 9 = diluted 10^{-2}, 10 = diluted 10^{-3}).

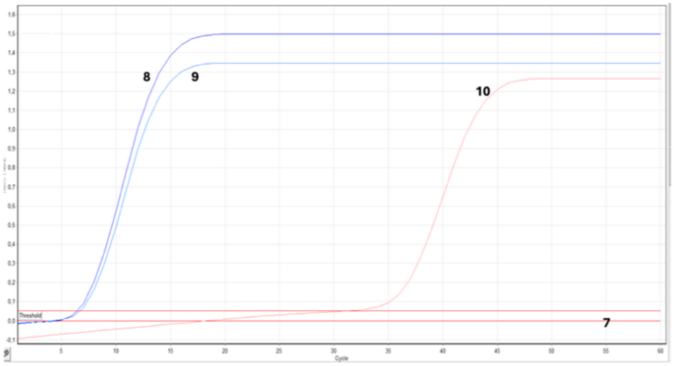

Supplement: Supplementary file 1 [file jof-12-00079-s001.zip › jof-4085582-supplementary.pdf]
